# Examining the influence of specificity ligands and ATP-competitive ligands on the overall effectiveness of bivalent kinase inhibitors

**DOI:** 10.1186/s12953-017-0125-1

**Published:** 2017-07-17

**Authors:** Margaret L. Wong, Jason Murphy, Edmund Harrington, Carrie M. Gower, Rishi K. Jain, Markus Schirle, Jason R. Thomas

**Affiliations:** 1 Novartis Institutes for BioMedical Research, Cambridge, MA 02139 USA; 2 Departments of Chemistry, University of Washington, Seattle, WA 98195 USA

**Keywords:** Chemical proteomics, Isobaric Tagging, Kinase profiling, Bivalent kinase inhibitor, SNAPtag, Structure activity relationship

## Abstract

**Background:**

Identifying selective kinase inhibitors remains a major challenge. The design of bivalent inhibitors provides a rational strategy for accessing potent and selective inhibitors. While bivalent kinase inhibitors have been successfully designed, no comprehensive assessment of affinity and selectivity for a series of bivalent inhibitors has been performed. Here, we present an evaluation of the structure activity relationship for bivalent kinase inhibitors targeting ABL1.

**Methods:**

Various SNAPtag constructs bearing different specificity ligands were expressed in vitro. Bivalent inhibitor formation was accomplished by synthesizing individual ATP-competitive kinase inhibitors containing a SNAPtag targeting moiety, enabling the spontaneous self-assembly of the bivalent inhibitor. Assembled bivalent inhibitors were incubated with K562 lysates, and then subjected to affinity enrichment using various ATP-competitive inhibitors immobilized to sepharose beads. Resulting eluents were analyzed using Tandem Mass Tag (TMT) labeling and two-dimensional liquid chromatography-tandem mass spectrometry (2D–LC-MS/MS). Relative binding affinity of the bivalent inhibitor was determined by calculating the concentration at which 50% of a given kinase remained bound to the affinity matrix.

**Results:**

The profiling of three parental ATP-competitive inhibitors and nine SNAPtag conjugates led to the identification of 349 kinase proteins. In all cases, the bivalent inhibitors exhibited enhanced binding affinity and selectivity for ABL1 when compared to the parental compound conjugated to SNAPtag alone. While the rank order of binding affinity could be predicted by considering the binding affinities of the individual specificity ligands, the resulting affinity of the assembled bivalent inhibitor was not predictable. The results from this study suggest that as the potency of the ATP-competitive ligand increases, the contribution of the specificity ligand towards the overall binding affinity of the bivalent inhibitor decreases. However, the affinity of the specificity components in its interaction with the target is essential for achieving selectivity.

**Conclusion:**

Through comprehensive chemical proteomic profiling, this work provides the first insight into the influence of ATP-competitive and specificity ligands binding to their intended target on a proteome-wide scale. The resulting data suggest a subtle interplay between the ATP-competitive and specificity ligands that cannot be accounted for by considering the specificity or affinity of the individual components alone.

**Electronic supplementary material:**

The online version of this article (doi:10.1186/s12953-017-0125-1) contains supplementary material, which is available to authorized users.

## Background

Small molecules are powerful tools for understanding complex biological systems. While the discovery of CRISPR is revolutionizing both the scale and precision of biological questions that can be addressed, small molecule approaches will continue to endure and complement such genetic techniques [1]. In some cases, the redundant functions of highly related proteins must be simultaneously inhibited in order to unveil a novel phenotypic effect [2]. In other cases, the binding of small molecules enhance catalytic efficiency by stabilizing molecular complexes [3]. Finally, in yet other cases, small molecules can bind to proteins to impart entirely new functions [4–6].

One of the central challenges in identifying small molecules suitable for use in cellular assays is achieving selectivity for the intended target. Sufficient selectivity is typically achieved through iterative rounds of carefully planned synthetic chemistry; an uncertain road with no guarantee of success. Bivalent inhibition is one strategy for rationally designing selective compounds [7]. In this design, a small molecule that modulates the function of a protein is linked to another molecule that has measurable affinity for the protein of interest, but binds at a secondary site. As a result of tethering the two binding modalities together, bivalent inhibitors exhibit enhanced binding affinity and selectivity for their intended target over the monovalent components.

Generally the design of bivalent inhibitors is carefully crafted with one specific target in mind; the design of each bivalent inhibitor is a unique solution for one specific target. One exception has been the development of SNAPtag-based bivalent kinase inhibitors. In this approach, SNAPtag serves as a linker between an ATP-competitive inhibitor and specificity ligand. Specificity ligands are expressed as fusions with SNAPtag. Small molecules are modified to contain a SNAPtag targeting element, which serves as a reactive group that enables the addition of the ATP-competitive inhibitor to the SNAPtag, thus enabling the spontaneous self-assembly of bivalent inhibitors. By keeping the SNAPtag portion constant but substituting different specificity ligands and/or different ATP-competitive inhibitors, SNAPtag-based bivalent inhibitors have been developed in such a modular fashion for SRC, ABL1, PIM1, MAPK14, EGFR, and phospho-MAPK1/3 [8–11]. Importantly, SNAPtag-based bivalent inhibitors have shown the ability to self-assemble in cells to modulate critical signaling pathways [11].

In order to aid in the development of future bivalent inhibitors, we sought to take advantage of the modularity of the SNAPtag strategy to dissect the contribution of the individual components on the overall binding affinity and resulting selectivity of the assembled bivalent. Using a chemical proteomic strategy to enrich kinases via ATP-competitive ligands conjugated to sepharose beads we assayed the ability of numerous SNAPtag-based bivalent kinase inhibitors, varying in potency and selectivity at both the ATP-competitive and specificity ligand, to engage their intended target as well as potential off-targets.

## Methods

See Additional file 1 for compound synthesis and characterization.

### Protein expression and purification

SNAPtag protein plasmids were transformed into BL21(DE3) *E. coli* cells and three colonies were used to inoculate LB broth (3 × 1000 mL) with carbenicillin (100 μg/mL). Cultures were grown at 37 °C to an OD_600_ of 0.6, cooled to 30 °C and induced with 1.0 mM IPTG (isopropyl β-D-1-thiogalactopyranoside). Proteins were expressed at 30 °C for 3.5 h. Cells were harvested by centrifugation (4000 rpm, 4 °C, 30 min), and the pellets were stored at −80 °C. For protein purification, the pellets (~4 g) were thawed at 0 °C and resuspended in lysis buffer (50 mM Tris pH 7.5, 100 mM NaCl, 10 mM imidazole) (25 mL) supplemented with 1× BugBuster, 1× HALT protease inhibitor cocktail, 1–5 mg/mL lysozyme, 25 units/mL benzonase. The suspension was incubated with gentle rocking at 4 °C until complete lysis was observed. The lysate was cleared by centrifugation (12000 rpm, 10 min, 4 °C). The cleared lysate was added to pre-equilibrated TALON Metal Affinity resin (3–5 mL) and rotated at 4 °C for 30 min. The resin was washed with lysis buffer (2 × 30 mL; no supplements), resuspended in lysis buffer (12 mL), and transferred to a column. SNAPtag-containing proteins were eluted with elution buffer (50 mM Tris pH 7.5, 100 mM NaCl, 200 mM imidazole). The most concentrated fractions were pooled, dialyzed into storage buffer (50 mM Tris, pH 7.5, 100 mM NaCl, 10% glycerol, 1 mM DTT), and concentrated using diafiltration units (MWCO 10000 Da). Proteins were analyzed by SDS-PAGE and found to be >95% pure by Coomassie stain. The proteins were separated into aliquots, snap-frozen and stored at −80 °C.

### Bivalent inhibitor assembly and purification

SNAPtag constructs were labeled with ATP-competitive-BG (o-benzylguanine) using the following conditions. Purified SNAPtag protein (100 μM) was incubated with ATP-competitive-BG (150 μM; 1.5-fold excess) in labeling buffer (20 mM Tris buffer, pH 8, 100 mM NaCl, and 1 mM DTT) for 1.5 h at 25 °C. Assembly reactions were monitored by intact protein mass spectrometry using a Waters Xevo G2-XS QToF MS instrument. If the reaction was incomplete, an additional 0.5–1.0 equivalent of ATP-competitive-BG was added. The protein-small molecule conjugates were then purified using GE Healthcare PD-10 Desalting Columns equilibrated with 50 mM HEPES pH 7.5, 150 mM NaCl, 1.5 mM MgCl_2_, 5% glycerol and 1 mM DTT. Labeling reactions were purified twice using two PD-10 Desalting Columns according to the manufacturer’s procedure. The concentration of the eluted protein was determined using the Pierce 660 nm Protein Assay Kit (Pierce Biotchencology). Constructs were snap-frozen and stored at −80 °C.

### Synthesis of ATP-competitive affinity matrix

For synthesis of KAM-derivatized resin, packed NHS-activated sepharose 4 fast flow resin (volume = 2 mL; GE Healthcare) was washed with anhydrous DMSO (3 × 10 mL). To the washed NHS-activated sepharose resin was added 0.5 mM KAM in anhydrous DMSO (8 mL; 2 μmol compound/mL of resin), followed by the addition of triethylamine (30 μL). The reaction mixture was vortexed to mix and pelleted by centrifugation (100 x g, 2 min). An aliquot of the supernatant (50 μL) was saved for LC/MS analysis. The reaction mixture was allowed incubate overnight at room temp with end-over-end rotating agitation. On the following day, the reaction mixture was pelleted by centrifugation (100 x g, 2 min). An aliquot of the supernatant (50 μL) was saved for LC/MS analysis. Completion of coupling was inferred by loss of starting material following LC/MS analysis. 2-(2-Aminoethoxy)ethanol (100 μL; Sigma-Aldrich) was added to the reaction mixture, vortexed, and incubate overnight at room temp with end-over-end agitation. The KAM-derivatized resin was then washed with anhydrous DMSO (3 × 10 mL) and 95% EtOH (3 × 10 mL).

For synthesis of imatinib-derivatized resin, a similar protocol was followed as described above except that final concentration of compound on the bead was 0.25 μmol compound/mL.

For synthesis of dasatinib-derivatized resin, the protocol for the KAM-derivatized resin was followed.

### K562 lysate generation

K562 cells were cultured in RPMI media supplemented with 10% fetal bovine serum and penicillin/streptomycin. Cells were incubated at 37 °C in a humidified atmosphere containing 5% CO_2_. K562 cell pellets were thawed on ice and resuspended in cold Lysis buffer (2× cell pellet volume; 50 mM HEPES pH 7.4, 150 mM NaCl, 1.5 mM MgCl_2_, 1 mM DTT, 0.8% NP40, 1× HALT protease inhibitor (Pierce Biotechnology)). The resuspended cell pellet was lysed using a dounce homogenizer (10 strokes with tight fitting pestle) and then pelleted by centrifugation (800 x g, 10 min., 4 °C). The resulting supernatant (S0.8) was stored on wet ice, while the pellet (P0.8) was processed further. The P0.8 pellet was first resuspended using cold Low Salt Buffer (0.5× pellet volume, 20 mM HEPES pH 7.4, 25% glycerol, 1.5 mM MgCl_2_, 0.2 mM EDTA, 1 mM DTT, 1× HALT protease inhibitor) and then cold High Salt Buffer (0.5× pellet volume, Low Salt Buffer +2.4 M NaCl) was added dropwise. The resuspended P0.8 pellet was further lysed via pressure cycling (Barocycler NEP2320, Pressure Biosciences Inc.) with 5 cycles of 35000 PSI for 20 s followed by atmospheric pressure for 20 s at 4 °C, and incubated overnight at 4 °C with benzonase (Sigma) at a final concentration of 90 units/mL. The resulting P0.8 lysate was pelleted by centrifugation (14000 x g, 20 min, 4 °C). The S0.8 and P0.8 lysates were combined and this combined lysate was used as input material for affinity enrichment experiments.

### Affinity enrichment and compound competition experiments in K562 lysates

For each affinity enrichment condition, 5 mg/mL K562 lysate (5 mg per treatment) was preincubated with either varying concentration of competition compound or DMSO control for 1 h at 4 °C. During this preincubation, the ATP-competitive-derivatized sepharose beads (35 μL per treatment) was washed (3 x with 3 mL) using Wash Buffer 2 (50 mM HEPES pH 7.4, 150 mM NaCl, 1.5 mM MgCl_2_, 1 mM DTT, 0.4% NP40). Preincubated lysates were then incubated with ATP-competitive-derivatized resin for 4 h at 4 °C with end-over-end agitation. The beads were transferred to individual columns (MoBiTec), washed with Wash Buffer 2 (3 mL; 50 mM HEPES pH 7.4, 150 mM NaCl, 1.5 mM MgCl_2_, 0.4% NP40, 1 mM DTT), Wash Buffer 1 (1.5 mL; 50 mM HEPES pH 7.4, 150 mM NaCl, 1.5 mM MgCl_2_, 1 mM DTT). To elute bound proteins, 2× LDS sample buffer (50 μL; NuPAGE) and 10 mM DTT was added to each sample, which were incubated at 55 °C for 30 min. Eluted proteins were separated from resin by centrifugation (14000 x g, 2 min, room temp). Proteins were alkylated with 200 mg/mL iodoacetamide for 30 min in the dark.

### Sample preparation and mass spectrometry data acquisition and analysis

Detergent was removed from the samples using detergent removal spin columns according to manufacturer’s protocol (Pierce Biotechnology). Proteins were subjected to in-solution trypsinization over night at 37 °C followed by isobaric labelling using either TMT 6-plex or TMT 10-Plex reagents (Thermo Fisher) using the labels 126–130 for varying concentrations of competitor compound and 131 for the DMSO-treated control sample. Samples were mixed and separated using high pH reverse phase chromatography (Dionex Ultimate 3000 HPLC, Waters Xbridge column (1 mm × 15 cm), mobile phase A: 100% H_2_O; mobile phase B: 100% AcN; mobile phase C (modifier, constant at 10%): 200 mM ammonium formate, pH 10; flow rate: 250 μL/min, 60 min effective gradient). Fractions were pooled to 16 samples which were analyzed by nanocapillary liquid chromatography-tandem mass spectrometry on an Easy-nLC 1000 HPLC system coupled to a Q-Exactive mass spectrometer (Thermo Scientific), using an in-house fabricated 75 μm ID spraying capillary packed with ReproSil-Pur 120 C18-AQ, 3 μm material (Dr. Maisch GmbH; 150 mm bed length) with a vented trapping column set-up (1 cm Michrom Magic C18AQ, 5 μm). The peptides were eluted with a gradient of 3% Buffer B (70% acetonitrile in 0.1% formic acid) to 45% B in 80 min (0.5%B/min) delivered at a flow rate of 300 nL/min and using a top 12 HCD data-dependent acquisition method. Peptide mass and fragmentation data were searched against a combined forward-reverse UniProt canonical human protein sequence database (version Jan 9 2013) supplemented with typical lab contaminants using Mascot (Matrix Science). Precursor and fragment ion tolerances were set to 10 ppm and 0.1 Da, respectively, allowing for 2 missed tryptic cleavages. Carbamidomethyl (C) was selected as fixed modification and TMT6 (K), TMT6 (N-term), Oxidation (M) as variable modifications. Peptide and protein validation was done using Transproteomic pipeline v3.3sqall (Institute for Systems Biology; http://tools.proteomecenter.org/software.php) using a false positive threshold of <1% for protein identifications. For each peptide sequence and modification state, reporter ion signal intensities from all spectral matches were summed for each reporter ion type and corrected according to the isotope correction factors given by the manufacturer. Only peptides unique to a given protein within the total dataset of identified proteins were used for relative protein quantification. Peptide fold changes were calculated (treatment over DMSO control) and subsequently renormalized within each experimental analysis using the median fold change of all quantified peptides to compensate for differences in total protein yield for each affinity purification. Protein fold changes were calculated as median peptide fold change (expressed on a Log10 scale) and *p*-values were calculated using a one-way T-test (arbitrarily set to 1 for nonsignificant single peptide quantitations) and adjusted using the Benjamini-Hochberg False Discovery Rate (FDR). Data were visualized for further analysis using Spotfire DXP. For calculating residual binding 50 (RB_50_) values (the point at which 50% of a given protein remained bound to the affinity matrix) resulting from competitor competition, for each protein the percent residual binding at each concentration was calculated (100*(1–10^Log10 fold change value)). XLfit was used for curve fitting and determination of RB_50_ values using a constrained 4 parameter single-site dose-response model (eq. 200): (A+(B/(1 + ((x/C)^D)))); where A is the low end of the curve fit (set = 0% residual binding), B is the high end of curve fit (set = 100% residual binding), and D is the Hill slope (set = 1).

## Results

**Fig. 1:**
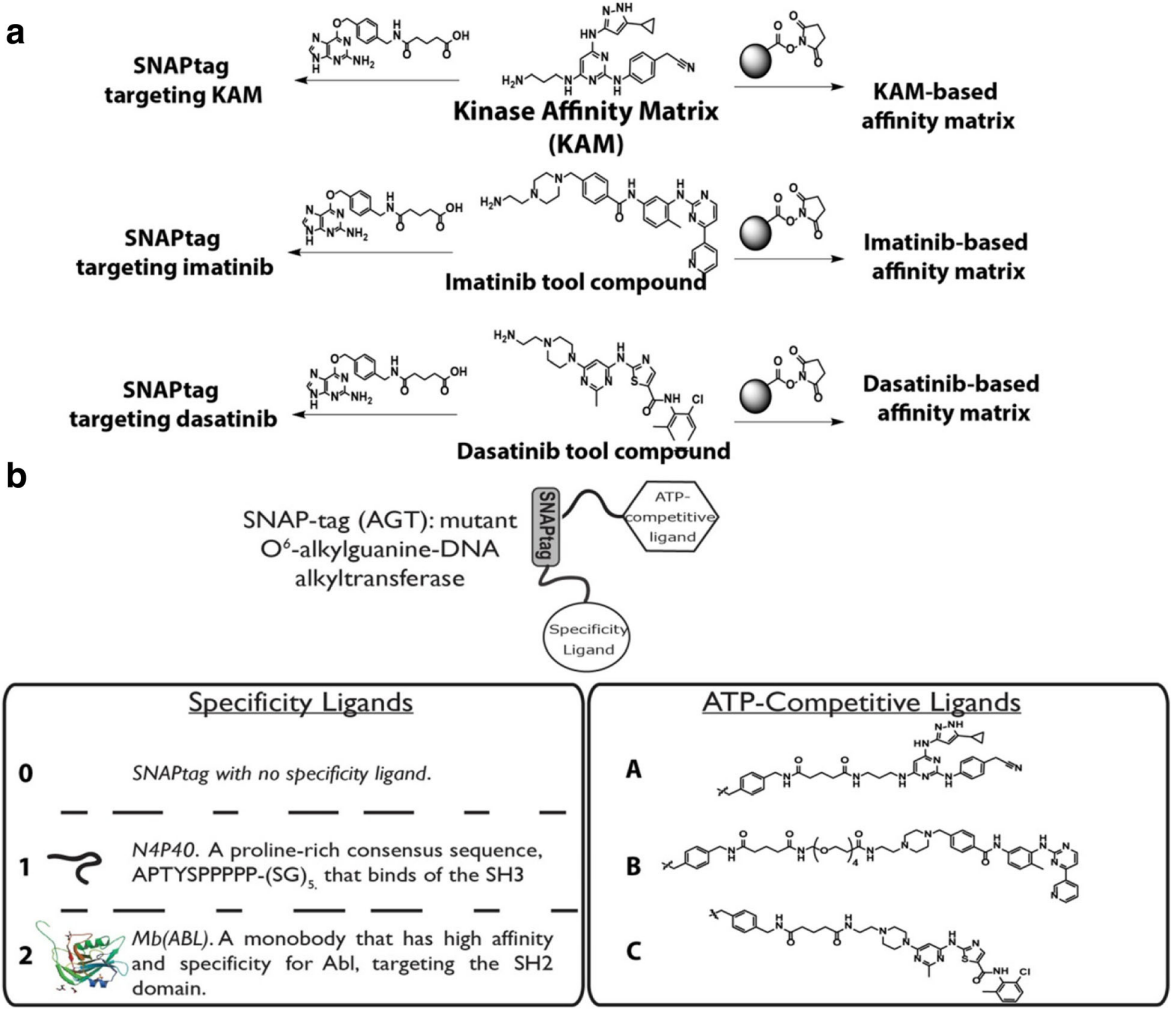
Compounds and reagents used in this study. **a** Versatile reagents based on kinase inhibitors facilitates rapid generation of SNAPtag targeting kinase inhibitor and affinity matrices. **KAM** has been previously described by our group as compound 3. **b** Combinatorial library of ABL1 targeting SNAPtag-based bivalent inhibitors. Using SNAPtag as a universal linker, various specificity ligands and ATP-competitive ligands can be displayed from SNAPtag and used in combination to evaluate the influence of each component towards ABL1 binding

To evaluate the contribution of each independent component on the overall potency and selectivity of the assembled inhibitor, we focused our attention on well validated ATP-competitive inhibitors and specificity ligands (see Fig. 1). While the potency and specificity of both the ATP-competitive inhibitors and specificity ligands vary considerably, common among them is the ability to bind to ABL1. By creating a small combinatorial library of SNAPtag-based bivalent inhibitors we aim to evaluate the on-target potency for ABL1 while assessing selectivity against potential off-targets present in K562 lysates.

Previously, we have reported **KAM** as a broad spectrum kinase inhibitor capable of profiling >200 endogenously expressed kinases [11]. Incubation of K562 lysates with a **KAM**-based affinity matrix enabled the identification of 229 kinases. AAK1 exhibited the greatest affinity for **KAM** (RB_50_ = 0.008 μM), while ABL1 exhibited much more modest affinity (RB_50_ = 0.759 μM) (see Table 1). Consistent with previous finding, conjugation of **A** to SNAPtag led to a significant decrease in overall affinity; an average 17-fold loss in potency was observed for kinases that were competed by preincubation with 10 μM **KAM**. It is presumed that the loss in potency is the result of steric hindrance. Addition of the specificity ligand **1** to SNAPtag afforded the bivalent inhibitor **A-1** which displayed comparable affinity for ABL1 as the parent compound (RB_50_ = 1.05 μM). While not improving the overall affinity **A-1** demonstrates greatly enhanced specificity for ABL1. **A-1** shows a 6-fold selectivity over the top off-target, AAK1 (RB_50_ = 1.05 μM vs. RB_50_ = 6.22 μM) and a 2.9-fold window of selectivity over the next most potently competed kinase GSK3B (RB_50_ = 3.07 μM). Consistent with previous reports, changing the specificity ligand to **2** provided bivalent inhibitor **A-2** that exhibited a marked ~10-fold enhanced potency for ABL1 (RB_50_ value = 0.070 μM vs. 0.759 μM) with a 79-fold window of selectivity over AAK1. Considering the contribution of the specificity ligands to the overall affinity of the bivalent inhibitor, the relative differences in the reported affinities of each of these specificity ligands suggests that **2** is ~40-fold more potent than **1**. The difference in potency of the assembled bivalent inhibitors is 15-fold and is largely reflective of the inherent differences in affinity of the specificity components.

**Table 1:** Assessment of affinity for **A**-series bivalent inhibitors for selected kinases

Gene Name	KAM RB_50_ (μM)	A-0 RB_50_ (μM)	A-1 RB_50_ (μM)	A-2 RB_50_ (μM)
ABL1	0.759	>10	1.05	0.070
AAK1	0.008	0.302	6.22	0.815
GSK3A	0.037	1.33	3.26	0.046
GSK3B	0.045	2.05	3.07	0.053
CDK9	0.112	9.03	>10	0.957
NEK9	0.086	5.77	>10	0.514

For affinity assessment of affinity for all kinases see Additional file 1

Imatinib is a well-known BCR-ABL inhibitor with high specificity but moderate affinity for BCR-ABL. Incubation of K562 with an imatinib-based affinity matrix led to the identification of 128 protein kinases, of which only BCR-ABL was competed by the addition of 10 μM imatinib (see Table 2). Consistent with the results observed with **A-0**, conjugation of **B** to SNAPtag led to a significant decrease in overall affinity; on average ~ 60-fold loss in potency was observed for proteins that were competed by preincubation with 10 μM imatinib. Addition of the specificity ligand **1** led to a bivalent inhibitor (**B-1**) with enhanced affinity for ABL1 (RB_50_ = 1.01 μM). Switching the specificity ligand to **2**, enhanced the potency by 13-fold (**B-2** RB_50_ = 0.106 μM). Consistent with the **A**-series of bivalent inhibitors, the rank order of affinity for the assembled bivalent inhibitors is reflected by the individual affinities of the specificity ligands. Even though **2** is already a selective BCR-ABL inhibitor, it is not without its off-targets. For example, the oxidoreductase NQO2 is commonly found as an off-target of many kinases inhibitors in lysate-based chemical proteomics experiments, including imatinib [12]. Addition of either specificity element completely eliminated NQO2 binding.

**Table 2:** Assessment of affinity for **B**-series bivalent inhibitors for proteins showing competition

Gene Name	Imatinib RB_50_ (μM)	B-0 RB_50_ (μM)	B-1 RB_50_ (μM)	B-2 RB_50_ (μM)
ABL1	0.107	6.45	1.01	0.106
NQO2	4.93	6.71	>10	>10

For affinity assessment of affinity for all kinases see Additional file 1

Dasatinib is a dual SRC/ABL inhibitor and has the greatest affinity for ABL1 of the inhibitors used in this study. Incubation of K562 lysates with a dasatinib-based affinity matrix enabled the identification of 81 protein kinases. SRC and ABL1 both displayed potent binding affinity for dasatinib (RB_50_ = 0.008 and 0.014 μM, respectively) (See Table 3). Conjugation of **C** to SNAPtag resulted in a substantial but less drastic reduction in overall binding affinity for its targets; on average the RB_50_ values were 9-fold weaker when compared to dasatinib alone. Addition of the specificity ligand **1** rescued the affinity for ABL1 back to levels of the parent compound (**C-1** RB_50_ = 0.029 μM). Switching the specificity ligand to **2** resulted in a bivalent inhibitor with only slightly better affinity (**C-2** RB_50_ = 0.019 μM). As is the case of the **A**- and **B**-based bivalent inhibitors the rank order of affinity of the assembled bivalent inhibitors is preserved based on the affinities of the individual components. However, the relative differences in affinity are decreased to the point where it is questionable whether there is a meaningful difference between them. Even though the overall potencies of the assembled bivalent inhibitors did not surpass that of the parent compound, it is important to note that the design of the bivalent inhibitors was successful; ABL1 was the most potently competed kinase for both **C-1** and **C-2**.

**Table 3:** Assessment of affinity for **C**-series bivalent inhibitors for selected kinases

Gene Name	Dasatinib RB_50_ (μM)	C-0 RB_50_ (μM)	C-1 RB_50_ (μM)	C-2 RB_50_ (μM)
ABL1	0.014	0.115	0.029	0.019
SRC	0.008	0.075	0.045	0.038
YES1	0.009	0.153	0.070	0.041
BTK	0.119	0.272	0.399	0.246
EPHB4	0.005	0.088	0.070	0.070
LYN	0.014	0.229	0.088	0.311

For affinity assessment of affinity for all kinases see Additional file 1

## Discussion

One of the appealing aspects of bivalent inhibitors as a strategy for improving the specificity and affinity of a starting compound is that it is based on the first principles of binding for the monovalent components. A rational bivalent inhibitor design is particularly beneficial when structural insight is not available for the target of interest to guide optimization of chemical matter.

From the prospective of first principles, it would be expected that the affinity of the assembled bivalent inhibitor would be dictated by the affinities of the ATP-competitive and specificity ligand. Perhaps the most unexpected observation from our data is that the contribution of the specificity ligand to the overall potency of the assembled bivalent inhibitor is variable and depends on the affinity of the ATP-competitive ligand. The binding affinity of **2** for the SH2 domain of ABL1 is 0.009 μM [13], while the binding affinity of **1** for the SH3 is ~0.4 μM [14]. Keeping the ATP-competitive portion constant and varying the specificity ligand resulted in a 15-fold, 10-fold, and 2-fold difference in RB_50_ values for ABL1. This suggests that as the affinity of the ATP-competitive ligand increases the contribution of the specificity ligand on overall binding affinity is diminished. While not entirely predictive, it is worth emphasizing that the most potent specificity ligand always resulted in the most potent assembled bivalent inhibitor within a given series.

From the outset of this study, one may have anticipated that the degree of specificity imparted by the specificity ligand would correlate with the binding affinity for ABL1. Addition of the weakest affinity specificity ligand **1**, regardless of the inherent specificity or affinity of the ATP-competitive ligand, consistently resulted in an assembled bivalent inhibitor with highest affinity for ABL1. These data suggest that even specificity ligands of modest affinity can be useful to achieving potent and selective bivalent inhibitors.

It is important to emphasize that while this study highlights the versatility of the SNAPtag-based bivalent inhibitor strategy, this approach is not without its limitations. Most notably, even though ATP-competitive ligands and specificity elements of modest potency can be combined to yield a bivalent kinase inhibitor with enhanced potency and selectivity, implementation of this strategy requires the identification of two ligands that bind at distinct sites. Here, promiscuous ATP-competitive ligands can be leveraged as starting points for a kinase target of interest and high-throughput selection strategies have the potential to yield novel secondary site ligands. However, even the most promiscuous kinase inhibitor is not capable of engaging every kinase and there is no guarantee of success with any selection screen.

In addition to providing the first structure activity relationship for a series of bivalent inhibitors against its target, this work also represents the most comprehensive selectivity profiling of bivalent kinase inhibitors. By applying an unbiased chemical proteomic profiling strategy we were able to simultaneously profile the relative affinity for ABL1 as well as 348 other kinases present in K562 lysates. Detailed analysis of the resulting data has enabled us to identify new potential off-targets of bivalent inhibitors that is not predicted by the profiles of the individual components. For example by comparing the resulting RB_50_ values for specific kinases within the **A**-series it is apparent that the affinities of GSK3B, GSK3A, CDK9, and NEK9 are enhanced for the bivalent inhibitor **A-2** (see Table 1). In our original characterization of **A-2**, there was a suggestion from the profiling data that these kinases indeed exhibited enhanced affinity for **A-2** relative to **A-0**. However, without a second specificity ligand targeting ABL1 it was not clear whether this result was due to direct binding. Based on the profiling data presented here we can now conclude that the enhanced RB_50_ values are unlikely to be the result of a protein-protein interaction network with ABL1, as the RB_50_ values for these putative additional targets is similar between **A-0** and **A-1.** These data suggest that the unique combination of **A** and **2** give rise to a bivalent inhibitor with enhanced potency for targets not predicted by considering the monovalent components alone. It is likely this ability of bivalent inhibitors to acquire affinity to targets, through unique binding distances and geometries, that are simply not available to the monovalent components that make predicting the affinity and selectivity of the assembled bivalent inhibitors difficult.

## Conclusion

The modular nature of SNAPtag-based bivalent kinase inhibitors and the thorough profiling afforded by chemical proteomics has enabled the first and most comprehensive evaluation of a structure activity relationship of bivalent kinase inhibitors. While anchored in the fundamental principles of binding energetics, the rules governing the contribution of each individual component are more complicated than understanding the respective selectivity and affinity of the monovalent components. More work is needed to understand some of the more subtle contributions of specific ATP-competitive and specificity ligand components. Fortunately, the modular nature of the SNAPtag-based bivalent inhibitors can enable rapid generation of even more bivalent kinase inhibitors to be evaluated through chemical proteomics. While this work has been exclusively focused on SNAPtag-based bivalent inhibitors, we believe that the results from this study are likely translatable to other non-SNAPtag bivalent inhibitor designs.

## Additional file

Additional file 1: Supporting mass spect data for protein kinases identified in the various chemical proteomic experiments. (ZIP 501 kb)

## Abbreviations

2D–LC-MS/MS: two-dimensional liquid chromatography-tandem mass spectrometry
BG: o-benzylguanine
RB_50_: 50% residual binding
TMT: Tandem Mass Tag
