# Supplementary material for: Examining the influence of specificity ligands and ATP-competitive ligands on the overall effectiveness of bivalent kinase inhibitors
Source: Proteome Sci. 2017 Jul 17;15:17. doi: 10.1186/s12953-017-0125-1 (PMC5513037; doi:10.1186/s12953-017-0125-1)
Supplement: Additional file 1: — Supporting mass spect data for protein kinases identified in the various chemical proteomic experiments. (ZIP 501 kb) [file 12953_2017_125_MOESM1_ESM.zip › Supporting Information_synthesis and characterization.doc]

**Materials and Methods**

Unless otherwise stated, all reagents were obtained from commercial suppliers and used without further purification.

^1^H NMR spectra were recorded on Bruker AV-400 (400 MHz),

LC/ESI-MS Data were recorded using a Waters LCT Premier mass spectrometer with dual electrospray ionization source and Agilent 1100 liquid chromatograph. HPLC separation was performed using 1.0 mL/min flow rate with the gradient from 5% to 95% ACN in 10 min, 0.1% Formic Acid used as the modifier additive in the Aqueous Phase. 3.0 x 100mm 3um C18 column.

^1^H NMR and ^13^C NMR spectra were recorded on Bruker Advance instruments. Data are reported

as follows: chemical shift in ppm, multiplicity (s = singlet, br s = broad singlet, d= doublet, t = triplet, q =

quartet, m = multiplet, dd = doublet of doublets), coupling constants *J* in Hz, and signals were referenced

against solvent peaks.

Preparative reverse-phase HPLC was performed on a Waters system equipped with Sunfire 5 μm

30 x 50 mm column with a gradient using 0.1% TFA in acetonitrile and H2O or an X-bridge 5 μm 30x50

mm column, 5 mM NH_4_OH in acetonitrile and water with a flow rate of 75 mL/min with 1.5 mL for the

injection volume. UPLC-MS measurements were conducted on a Waters Acquity UPLC-UV-ELSDMS/

Waters SQD mass spectrometer system using a BEH column 1.7um 2.1 x 50mm at 50^o^ C with a gradient

comprised of the following eluents: [Eluent A: Water + 0.05% formic acid + 3.75mM Ammonium

Acetate], [Eluent B: Acetonitrile + 0.04% formic acid]; 2%-98% Eluent A 🡪 B in 4.4 min, 1 mL/min

flow rate. LC-UV/HRMS was measured on an Waters Acquity G2 Xevo QTof, using a CSH column 1.7

um 2.1 x 50mm at 50^o^ C, with a gradient comprised of the following eluents: [Eluent A = Water + 0.1%

Formic Acid + 3.75mM Ammonium Acetate], [Eluent B: Acetonitrile + 0.08% Formic Acid]. A gradient

of Eluent A 🡪B starting from 1%-30% in 3.2 min and then to 98% in another 1.95 min with a 1.0 mL/

min flow rate was used.

**Synthesis of compound 2:** A suspension of [**1**](http://smreg.nibr.novartis.net/smr/Lookup/NVP-MAN016-NX-1) (366 mg, 1.354 mmol) in dimethylacetamide (Volume: 9.0 mL) was sonicated for 5-10 minutes at 40-50 °C. Triethylamine (0.377 mL, 2.71 mmol), glutaric anhydride (155 mg, 1.354 mmol)and DMAP (74.4 mg, 0.609 mmol) were introduced. The reaction was stirred overnight at room temperature. The reaction mixture was diluted in water and acidified to pH 4 using aqueous HCl. The white precipitate was dried to obtain compound **2** (476 mg, 1.238 mmol, 91 % yield). 

1H NMR (z400 MHz, DMSO-d6) δ 12.41 (s, 1H), 12.03 (s, 1H), 8.32 (t, J = 5.7 Hz, 1H), 7.80 (s, 1H), 7.45 (d, J = 7.9 Hz, 2H), 7.26 (d, J = 8.0 Hz, 2H), 6.28 (s, 1H), 5.45 (s, 2H), 4.26 (d, J = 5.8 Hz, 2H), 2.22 (t, *J* = 7.4 Hz, 2H), 2.17 (t, J = 7.4 Hz, 2H), 1.77-1.70 (m, 2H). LCMS (ES+) [M+H]+ = (calculated) 385.2, (measured) 385.3. 

**Synthesis of compound 4:**  A suspension/solution of 6-methyl-N1-(4-(pyridin-3-yl)pyrimidin-2-yl)benzene-1,3-diamine **3**(268 mg, 0.966 mmol) and triethylamine (270 µL, 0.966 mmol) in toluene (Volume: 4.8 mL)was stirred at 4 C for ~10 min. 4-(chloromethyl)benzoyl chloride (210 mg, 1.111 mmol)  was introduced. The reaction was stirred at 4^0^C for 3 h. The yellow reaction mixture was added dropwise to vigorously stirring water. The resulting mixture was allowed to stir for 30-60 minutes. The yellow solid was collected by vacuum filtration and dried on high vac. 

An aliquot of the reaction was analyzed by LCMS; a single peak with m/z 430.1 was observed. The LCMS trace and m/z matched that of compound **4**. 

**Synthesis of compound 5:** Compound **4** (100 mg, 0.233 mmol), tert-butyl (2-(piperazin-1-yl)ethyl)carbamate (167. mg, 0.728 mmol) and DIEA (0.085 mL, 0.488 mmol) were suspended in 1,4-Dioxane (Volume: 2.4 mL) in a capped 5 mL conical microwave reaction vial. The mixture was heated by MW 170^0^ C for 10 sec with a 60 sec pre-stir. The majority of the solid mixture had converted to a amber colored solution with some solid remaining. The mixture was heated again by MW 170^0^C for 10 min with a 60 sec pre-stir. Some dark amber colored solids formed and adhered to the glass vial. The remaining solution was transferred to a 50 mL roundbottom flask. The solution was concentrated to afford an orange colored solid. The solid was transferred to a Buchener funnel and was triturated with water (1 x 5 mL), methanol (10-15 mL) and EtOAc (5 mL). The methanolic and ethyl acetate filtrates were combined and concentrated in vacuo to afford a viscous organge oil (~289 mg) . Crude compound was dissolved in CH2Cl2 (15 mL) and washed with H2O. Back extracted aq. phase with EtOAc (5 mL). Some product remained in aqueous phase. Extracted the aqueous phase again with CH2Cl2 (1 x 10 mL). The combined organic phases were dried over MgSO4 and concentrated in vacuo to afford a yellow solid that contained compound **5** (126.4 mg, 0.203 mmol, 87 % yield) and tert-butyl (2-(piperazin-1-yl)ethyl)carbamate (46.6 mg, 0.203 mmol, 87 % yield). 

*1H NMR* (400 MHz, DMSO-d6) δ 10.16 (s, 1H), 9.26 (d, J = 2.4 Hz, 1H), 8.99 (s, 1H), 8.70 - 8.64 (m, 1H), 8.53 - 8.43 (m, 2H), 8.10 - 8.03 (m, 1H), 7.89 (s, 1H), 7.87 (s, 1H), 7.54 - 7.7.49 (m, 1H), 7.48 - 7.44 (m, 1H), 7.44 - 7.39 (m, 3H), 7.19 (d, J = 8.3 Hz, 1H), 6.66 - 6.62 (m, 1H), 3.51 (s, 2H), 3.05 - 2.94 (m, 4H), 2.79 (t, J = 4.9 Hz, 3H), 2.43 - 2.23 (m, 14H), 2.20 (s, 3H), 1.35 (d, J = 2.2 Hz, 18H). 

**Synthesis of compound 6:** Compound **5** and tert-butyl (2-(piperazin-1-yl)ethyl)carbamate were used as a 1:1 mixture (173 mg) for the starting material of compound **6** without additional purification. A freshly prepared solution of CH2Cl2 (Volume: 4.4 mL) and trifluoroacetic acid (1.564 mL, 20.30 mmol) was added to compound **5** (126.4 mg, 0.203 mmol). 
After 15 min, the reaction solution was coevaporated with toluene (3 x 10-15 mL) to facilitate the azeotropic removal of TFA to afford an oil. The oil was diluted in methanol, loaded onto a disposable Strong Cation Exchange ("SCX) column, which had been previously washed with CH2Cl2 and methanol, and eluted with 2 N ammomnia in methanol to afford compound **6** (76 mg, 0.145 mmol, 71.6 % yield) and 2-(piperazin-1-yl)ethan-1-amine (18.8 mg, 0.146 mmol, 71.7 % yield) as a mixture (95 mg; 1:1 from NMR) 

*1H NMR* (400 MHz, Methanol-*d*4) δ 9.27 (d, *J* = 2.3 Hz, 1H), 8.62 (dd, *J* = 4.8, 1.6 Hz, 1H), 8.58 (dt, *J* = 8.0, 1.9 Hz, 1H), 8.45 (d, *J* = 5.2 Hz, 1H), 8.21 (d, *J* = 2.2 Hz, 1H), 7.90 (d, *J* = 8.2 Hz, 2H), 7.53 (ddd, *J* = 8.0, 4.9, 0.9 Hz, 1H), 7.51 - 7.44 (m, 2H), 7.40 (dd, *J* = 8.2, 2.2 Hz, 1H), 7.34 (d, *J* = 5.3 Hz, 1H), 7.24 (d, *J* = 8.3 Hz, 1H), 3.60 (s, 2H), 2.88 (d, *J* = 4.8 Hz, 9H), 2.58 - 2.41 (m, 15H), 2.30 (s, 3H). *LCMS* (QTof, ES+) C30H34N8O: [M+2H]2+ = (measured) 262.3. 

**Synthesis of compound 7:** A solution of 2,2-dimethyl-4-oxo-3,8,11,14,17-pentaoxa-5-azaicosan-20-oic acid (44.0 mg, 0.121 mmol), DIEA (48.4 µl, 0.277 mmol) and HATU (50.4 mg, 0.133 mmol) in DMF (Volume: 700 µl) was stirred at rt for 5-10 minutes after which the color of the solution gradually changed from colorless to yellow to dark yellow. This solution was added to an orange-yellow colored residue of compound **6** (63 mg, 0.121 mmol). The resulting amber colored solution was stirred at rt under N­­_2_ (g) for 2.5 days. The reaction solution was diluted with MeOH to a final volume of 3 mL, filtered through a syringe-driven filter and purified by preparative SFC (MS triggered @ m/z 870, 893 and 385; Method M1005: c:\masslynx\2014ms100_250.pro\acqudb\m1005. Two sets of fractions were collected (Product 001, RT = 4.09 - 4.94 min, m/z = 849, 870 and Product 002, RT = 4.94-6.67 min, m/z = 385, 870) to afford compound **7** (21 mg, 0.024 mmol, 20.02 % yield). 

1H NMR (400 MHz, Methanol-d4) δ 9.26 (dd, J = 2.3, 0.9 Hz, 1H), 8.62 (dd, J = 4.9, 1.6 Hz, 1H), 8.58 (ddd, J = 8.1, 2.3, 1.6 Hz, 1H), 8.45 (d, J = 5.2 Hz, 1H), 8.21 (d, J = 2.2 Hz, 1H), 7.95 - 7.87 (m, 2H), 7.53 (ddd, J = 8.1, 4.9, 0.9 Hz, 1H), 7.50 - 7.44 (m, 2H), 7.40 (dd, J = 8.2, 2.2 Hz, 1H), 7.34 (d, J = 5.2 Hz, 1H), 7.24 (d, J = 8.3 Hz, 1H), 3.72 (dt, J = 10.0, 6.2 Hz, 2H), 3.67 - 3.54 (m, 14H), 3.48 (t, J = 5.6 Hz, 2H), 3.34 (d, J = 7.1 Hz, 2H), 3.20 (t, J = 5.6 Hz, 2H), 2.68 - 2.46 (m, 9H), 2.43 (t, J = 6.1 Hz, 9H), 2.31 (s, 3H), 1.42 (s, 9H). 

**Synthesis of compound 8:** A 1:2 solution of trifluoroacetic acid (0.2 mL, 2.60 mmol) and CH2Cl2 (Volume: 0.4 mL) was added to compound **7** (21 mg, 0.024 mmol). After 30 minutes of reaction time, the reaction solution was partially concentrated to remove CH2Cl2. Methanol was added to facilitate the partial azeotropic removal of residual TFA. This crude product (30 mg) was dissolved in methanol and passed through  a disposable bicarbonate column that had been prewashed with methanol. The filtrate was concentrated in vacuo to afford compound **8** (21.5 mg, 0.028 mmol, 116 % yield). 

**Results** 
*1H NMR* (400 MHz, Methanol-d4) δ 9.26 (dd, J = 2.2, 0.9 Hz, 1H), 8.61 (dd, J = 4.9, 1.6 Hz, 1H), 8.57 (ddd, J = 8.1, 2.3, 1.7 Hz, 1H), 8.45 (d, J = 5.2 Hz, 1H), 8.21 (d, J = 2.2 Hz, 1H), 7.92 - 7.88 (m, 2H), 7.52 (ddd, J = 8.1, 4.9, 0.9 Hz, 1H), 7.48 - 7.44 (m, 2H), 7.39 (dd, J = 8.3, 2.2 Hz, 1H), 7.33 (d, J = 5.2 Hz, 1H), 7.24 (d, J = 8.0 Hz, 1H), 3.70 (t, J = 6.1 Hz, 2H), 3.63 - 3.57 (m, 14H), 3.53 (t, J = 5.3 Hz, 2H), 2.86 - 2.78 (m, 2H), 2.58 2.47 (m, 8H), 2.43 (t, J = 6.1 Hz, 2H), 2.30 (s, 3H). 

*13C NMR* (101 MHz, MeOD) δ 172.54, 167.02, 162.29, 161.22, 158.92, 150.39, 147.64, 141.37, 137.65, 136.81, 135.39, 134.00, 133.14, 130.17, 129.23, 127.74, 127.28, 123.97, 117.25, 116.94, 107.34, 70.96, 70.11, 70.09, 70.07, 70.04, 69.92, 69.78, 66.86, 61.95, 56.68, 52.54, 52.36, 48.45, 40.40, 36.22, 36.10, 16.47. 

**Synthesis of compound 9:** Compound **2** (8 mg, 0.021 mmol) and DMF (Volume: 200 µL) were added to a residue of compound **8** (16 mg, 0.021 mmol). DIEA (22 µL, 0.126 mmol) was introduced, and the white suspension was stirred at rt for 5 minutes. T3P, 50% in DMF (22 µL, 0.033 mmol) was added dropwise, and the resulting solution was allowed to stir at rt. After 4 h, the reaction was diluted in methanol (final volume = 3 mL) and filtered through a 0.45-um syringe filter. The filtrate was purified by preparative SFC (Method 4105: Princeton DEAP 20x150 mm 5 ìm column, CO2/MeOH) to afford compound **9** (13.2 mg, 10.57 µmol, 50.9 % yield). The product eluted over a broad window (RT = 2.89 min - 4.89 min). 

*HRMS* (QT1, Product Analysis_HRMS_Acidic.olp, FCA LCMS-ApH): C59H73N15O9, [M+H]+ = (calculated) , 1136.5796, (measured) 1136.5803; [M+2H]2+ = (calculated) 568.7938, (measured) 568.7908; [M+3H]3+ = (calculated) 379.5319, (measured) 379.5282. 

*1H NMR (400 MHz, Methanol-d4)* δ 9.26 (s, 1H), 8.61 (d, J = 3.8 Hz, 1H), 8.56 (dt, J = 8.0, 1.7 Hz, 1H), 8.44 (d, J = 5.2 Hz, 1H), 8.22 (d, J = 2.1 Hz, 1H), 7.98 - 7.89 (m, 2H), 7.83 (s, 1H), 7.56 - 7.43 (m, 5H), 7.40 (dd, J = 8.2, 2.2 Hz, 1H), 7.33 (d, J = 5.2 Hz, 1H), 7.25 (dd, J = 11.1, 8.4 Hz, 3H), 5.50 (s, 2H), 4.34 (s, 2H), 3.78 (d, J = 9.4 Hz, 2H), 3.70 (dd, J = 7.0, 5.0 Hz, 2H), 3.62 - 3.54 (m, 14H), 3.49 (t, J = 5.5 Hz, 2H), 3.44 (d, J = 5.9 Hz, 1H), 3.32 (s, 2H), 3.24 - 3.17 (m, 1H), 3.04 (s, 2H), 2.92 (s, 2H), 2.78 (s, 2H), 2.44 (t, J = 6.0 Hz, 2H), 2.30 (s, 3H), 2.23 (ddd, J = 11.2, 9.4, 5.8 Hz, 4H), 1.90 (q, J = 7.4 Hz, 2H), 1.67 (s, 2H), 1.40 - 1.23 (m, 3H), 1.02 (dt, J = 9.8, 5.5 Hz, 3H). 

*13C NMR (101 MHz, MeOD)* δ 174.01, 173.78, 166.79, 162.32, 161.23, 158.95, 150.40, 147.67, 138.74, 137.68, 136.74, 135.52, 135.40, 134.66, 133.14, 130.21, 129.47, 129.20, 128.37, 127.88, 127.58, 127.40, 127.28, 123.99, 117.31, 117.02, 107.37, 70.12, 70.09, 70.04, 69.98, 69.90, 69.82, 69.12, 67.25, 66.67, 60.76, 56.44, 51.76, 50.62, 42.43, 38.92, 36.10, 35.08, 34.80, 34.76, 21.84, 16.46, 7.77. 

**Synthesis of compound 10:** DIEA (15.99 µl, 0.092 mmol) was added to a solution of compound **2** (16 mg, 0.042 mmol) in dmf (Volume: 210 µl). After 5 min of stirring at room temperature, HATU (17.41 mg, 0.046 mmol). After another 5 min of stirring,compound **6** (22 mg, 0.042 mmol) was introduced. The reaction progress was assessed by LCMS after 1 h: product was present, though not clearly to be the major component of the reaction solution. The reaction was allowed to stir overnight at room temperature. The reaction solution was diluted in a mixture of methanol and DMSO (total volume = ~ 3 mL), passed through a syringe-driven filter and purified by preparative HPLC (2 x 1.5 mL injection; Method 3_basic: X-Bridge 30x50 mm 5 µm column with ACN/H2O with 5 mM NH4OH; 75 mL/min flow rate; 15-40% ACN 3.5 min gradient) to afford compound **10** (RT = 3.28 - 3.64 min) over two runs. A portion of the product (1.7 mg, 1.912 µmol, 4.59 % yield), was assayed by NMR and LCMS. 

**Results** 
1H NMR (400 MHz, DMSO-d6) δ 10.19 (s, 1H), 9.28 (d, J = 2.2 Hz, 1H), 9.01 (s, 1H), 8.69 (dd, J = 4.8, 1.7 Hz, 1H), 8.55 - 8.44 (m, 2H), 8.32 (t, J = 5.9 Hz, 1H), 8.08 (d, J = 2.1 Hz, 1H), 7.98 - 7.87 (m, 2H), 7.83 (s, 1H), 7.73 (t, J = 5.6 Hz, 1H), 7.52 (dd, J = 8.0, 4.8 Hz, 1H), 7.47 (d, J = 2.3 Hz, 1H), 7.44 (td, J = 8.2, 3.7 Hz, 5H), 7.26 (d, J = 8.0 Hz, 2H), 7.21 (d, J = 8.4 Hz, 1H), 6.29 (s, 2H), 5.45 (s, 2H), 4.26 (d, J = 5.8 Hz, 2H), 3.51 (s, 3H), 3.13 (q, J = 6.5 Hz, 2H), 2.89 (s, 1H), 2.73 (s, 1H), 2.47 - 2.27 (m, 12H), 2.22 (s, 3H), 2.13 (t, J = 7.5 Hz, 4H), 2.06 (t, J = 7.5 Hz, 2H), 1.77 - 1.69 (m, 2H). 

13C NMR (101 MHz, DMSO) δ 171.69, 171.54, 165.22, 162.27, 161.53, 161.11, 159.54, 159.45, 151.35, 148.15, 141.99, 139.49, 137.73, 137.15, 135.13, 134.38, 133.70, 132.14, 129.99, 128.60, 128.52, 127.56, 127.53, 127.20, 123.76, 117.12, 116.64, 107.46, 66.47, 61.58, 57.05, 52.71, 52.58, 41.75, 40.34, 36.01, 35.75, 34.75, 34.64, 30.72, 21.52, 17.64. 

HRMS [M+H]+ = (measured) 889.4357; [M+2H]2+ = (measured) 445.2199. RT = 1.17 min.

**Synthesis of compound 11:** 2-((6-chloro-2-methylpyrimidin-4-yl)amino)-N-(2-chloro-6-methylphenyl)thiazole-5-carboxamide (122 mg, 0.309 mmol), tert-butyl (2-(piperazin-1-yl)ethyl)carbamate (222 mg, 0.968 mmol), DIEA (0.108 mL, 0.619 mmol) and 1,4-dioxane (Volume: 1.0 mL) were added to a 5 mL conical microwave reaction vial. The mixture was heated by MW 160 C for 10 min with 30 sec pre-stir.  By LCMS SQ1 RXN MON Nonpolar), some starting material remained. A second microwave reaction was set up (MW170 for 12 min with 1-3 min pre-stir), but was halted after 1 few minutes of reaction time. The white mixture was transferred to a round-bottom flask, rinsing w EtOAc and concentrated to afford a peach-colored solid. The solid was triturated with water, methanol and diethyl ether. The solid was dried on high vac. A proton NMR (d6-DMSO) was acquired for compound **12**. 

1H NMR (400 MHz, DMSO-d6) δ 11.46 (s, 1H), 9.87 (s, 1H), 8.22 (s, 1H), 7.43 - 7.36 (m, 1H), 7.32 - 7.21 (m, 2H), 6.68 (s, 1H), 6.05 (s, 1H), 3.57 (s, 4H), 3.50 (s, 4H), 3.07 (q, J = 6.6 Hz, 2H), 2.45 (s, 4H), 2.40 (s, 3H), 2.36 (t, J = 6.8 Hz, 2H), 2.24 (s, 3H), 1.38 (s, 9H). 

**Synthesis of compound 13:** To a solution of compound **12** (115 mg, 0.196 mmol) in CH2Cl2 (Volume: 3 mL) was added trifluoroacetic acid (1.509 mL, 19.59 mmol). After 15-30 min, the reaction was concentrated in vacuo, using toluene to azeotropically co-evaporate the trifluoroacetic acid. 
The crude product was dissolved in aqueous DMSO (Volumetotal = 9 mL) and passed through a 0.4 um syringe-driven filter. The product was purified by preparative HPLC (Method 2_acidic: Sunfire 30x50 mm 5 µm column with 0.1% formic acid; 75 mL/ min flow rate; 10-30% ACN 3.5 min gradient) to afford compound **13** (91.4 mg, 0.138 mmol, 70.2 % yield)(RT  = 2.2 -2.8 min). 

*1H NMR* (400 MHz, DMSO-d6) δ 11.49 (s, 1H), 9.89 (s, 1H), 8.23 (s, 1H), 7.44 - 7.37 (m, 1H), 7.33 - 7.21 (m, 2H), 6.08 (s, 1H), 3.55 (s, 4H), 3.33 (s, 4H), 2.96 (d, J = 5.5 Hz, 2H), 2.55 (d, J = 5.7 Hz, 3H), 2.41 (s, 3H), 2.24 (s, 3H). DMSO signal overlaps with multiplets corresponding to C**H2**-C**H2**-NH2.

*13C NMR* (101 MHz, DMSO) ä 165.17, 163.06, 162.48, 162.28, 159.86, 158.31, 158.00, 157.70, 157.39, 156.91, 140.76, 138.77, 133.44, 132.37, 129.01, 128.17, 126.99, 125.70, 82.61, 54.23, 51.95, 43.38, 35.68, 25.57, 18.27. *HRMS*: (QT1, ES+) C22H27ClN8OS, [M+H]+ = (calculated) 487.1725, (measured) 487.1771. 

**Synthesis of compound 14:** DIEA (22.39 µl, 0.128 mmol) was added to a solution of compound **2** (22.4 mg, 0.058 mmol) in DMF (Volume: 146 µl). After 5 min of stirring at room temperature, HATU (24.37 mg, 0.064 mmol). After another 5 min of stirring,compound **13** (169 µl, 0.068 mmol) was introduced. The reaction progress was assessed by LCMS after 1 h: product was present and may be the major component of the reaction solution. The reaction was allowed to stir overnight at room temperature as this was shown to not be detrimental for similar reactions.  The reaction solution was diluted in a mixture of methanol and DMSO (total volume = ~ 3 mL), passed through a syringe-driven filter and purified by preparative HPLC (2 x 1.5 mL injection; Method 4_basic: X-Bridge 30x50 mm 5 µm column with ACN/H2O with 5 mM NH4OH; 75 mL/min flow rate; 25-50% ACN 3.5 min gradient) to afford compound **14** (RT = 2.24-2.60 min) over two runs. A portion of the product, compound **14** (2 mg, 2.344 µmol, 4.02 % yield)was assayed by NMR and LCMS. 

*1H NMR* (400 MHz, DMSO-d6) δ 8.33 (t, J = 5.9 Hz, 1H), 8.23 (s, 1H), 7.96 (s, 1H), 7.83 (s, 2H), 7.80 (t, J = 5.6 Hz, 2H), 7.49 - 7.43 (m, 3H), 7.41 (dd, J = 7.6, 1.9 Hz, 1H), 7.27 (dq, J = 7.3, 5.1, 4.3 Hz, 4H), 6.30 (s, 2H), 6.05 (s, 1H), 5.45 (s, 2H), 4.26 (d, J = 5.8 Hz, 2H), 3.55 - 3.47 (m, 4H), 3.19 (q, J = 6.5 Hz, 3H), 2.45 (t, J = 5.1 Hz, 13H), 2.41 (s, 3H), 2.38 (d, J = 6.9 Hz, 2H), 2.24 (s, 3H), 2.14 (t, J = 7.5 Hz, 4H), 2.08 (t, J = 7.5 Hz, 2H), 1.74 (p, J = 7.9, 7.5 Hz, 2H). 

*13C NMR* (101 MHz, DMSO) δ 171.69, 171.61, 165.11, 162.54, 162.29, 162.26, 161.07, 159.87, 159.56, 156.90, 140.79, 139.50, 138.77, 135.13, 133.46, 132.37, 128.99, 128.53, 128.14, 127.20, 126.97, 125.60, 82.59, 66.47, 56.99, 52.24, 43.50, 41.75, 40.34, 35.94, 35.75, 34.78, 34.63, 30.72, 25.56, 21.52, 18.28. 

HRMS [M+H]+ = (calculated) 853.3237, (measured) 853.3219. RT = 1.34 min. 

**Synthesis of compound 15:** (27.0 µl, 0.155 mmol) was added to a solution of compound **2** (27 mg, 0.070 mmol) in DMF (Volume: 350 µl). After 5 min of stirring at room temperature, HATU (29.4 mg, 0.077 mmol). After another 5 min of stirring, compound **19** (35 mg, 0.087 mmol) was introduced. The reaction progress was assessed by LCMS after 1 h: product was present and may be the major component of the reaction solution. The reaction was allowed to stir overnight at room temperature. The reaction solution was diluted in a mixture of methanol and DMSO (total volume = ~ 3 mL), passed through a syringe-driven filter and purified by preparative HPLC (2 x 1.5 mL injection; Method 3_basic:X-Bridge 30x50 mm 5 µm column with ACN/H2O with 5 mM NH4OH; 75 mL/min flow rate; 15-40% ACN 3.5 min gradient) to afford compound **15** (RT = 3.36 - 3.72 min) over two runs. A portion of the product, compound **15** (5 mg, 6.49 µmol, 9.25 % yield) was assayed by NMR and HRMS. 

**Results** 
*1H NMR* (400 MHz, DMSO-d6) δ 8.32 (t, J = 5.8 Hz, 1H), 7.95 (s, 2H), 7.81 (dd, J = 13.3, 6.7 Hz, 4H), 7.45 (d, J = 8.0 Hz, 2H), 7.25 (d, J = 8.0 Hz, 2H), 7.19 (d, J = 8.0 Hz, 2H), 6.32 (d, J = 15.4 Hz, 2H), 5.44 (s, 2H), 4.25 (d, J = 5.7 Hz, 2H), 3.92 (s, 2H), 3.36 (s, 8H), 3.22 (s, 1H), 3.11 (q, J = 6.4 Hz, 2H), 2.13 (t, J = 7.4 Hz, 2H), 2.07 (t, J = 7.4 Hz, 2H), 1.77 - 1.71 (m, 2H), 1.68 - 1.62 (m, 2H), 0.94 (d, J = 6.5 Hz, 3H), 0.87 (s, 2H), 0.68 - 0.62 (m, 2H). 

*13C NMR* (101 MHz, DMSO) δ 171.68, 171.62, 162.27, 159.77, 159.59, 155.12, 149.25, 145.20, 141.20, 139.46, 137.75, 135.14, 128.52, 127.91, 127.19, 118.70, 113.41, 92.29, 66.45, 41.75, 36.36, 35.75, 34.79, 34.68, 30.72, 21.67, 21.53, 7.67, 6.78, 0.08. 

HRMS [M+H]+ = (calculated) 770.3681, (measured) 770.3735; [M+2H]2+ = (calculated) 385.6844, (measured) 385.6902. RT = 1.28 min. 

**Synthesis of compound 16:** HATU (15.02 mg, 0.040 mmol) was added to a solution of DIEA (13.80 µl, 0.079 mmol) and **JQ-1** (carboxylic acid) (14.4 mg, 0.036 mmol) in DMF (Volume: 180 µl). After 5 minutes, a 0.5 M solution of tert-butyl (3-(2-(2-(3-aminopropoxy)ethoxy)ethoxy)propyl)carbamate (136 µl, 0.068 mmol) in DMF was introduced. The reaction was allowed to stir at rt overnight. The orange-colored reaction solution was diluted with brine and extracted with ~9:1 EtOAc-heptanes solution (2 x 2 mL). The combined organic phases were dried over MgSO_4_ and concentrated in the presence of Celite. The crude product that had been adsorbed onto Celite was purified by Isco (1-10% MeOH in CH_2_Cl_2_) to afford compound **17** (25 mg, 0.036 mmol, 99 % yield).

^1^H NMR (400 MHz, Methanol-*d*_4_) δ 7.43 (q, *J* = 8.7 Hz, 4H), 4.63 (dd, *J* = 8.8, 5.4 Hz, 1H), 3.69 – 3.54 (m, 11H), 3.49 (t, *J* = 6.3 Hz, 3H), 3.43 – 3.24 (m, 9H), 3.10 (t, *J* = 6.8 Hz, 2H), 2.69 (s, 3H), 2.45 (s, 3H), 1.83 (p, *J* = 6.4 Hz, 2H), 1.71 (d, *J* = 6.7 Hz, 5H), 1.42 (s, 10H).

**Synthesis of compound 17:** A freshly made solution of trifluoroacetic acid (250 µl, 3.24 mmol) and dichloromethane (500 uL) were added to the residue of compound **16** (25 mg, 0.036 mmol). After 15 mintues, the yellow-colored reaction appeared to have gone to completion by LCMS. The reaction solution was diluted in toluene and concentrated to remove the majority of the TFA and DCM (2 x 12 mL). The residue was dissolved in methanol and passed over a solid supported bicarbonate resin column to remove residual TFA, washing with methanol until the compound fully eluted, as observed by TLC. The filtrate was concentrated in vacuo to compound **17** (10.2 mg, 0.016 mmol, 46.1 % yield). 


1H NMR (400 MHz, Methanol-*d*4) δ 7.43 (q, *J* = 8.7 Hz, 4H), 4.63 (dd, *J* = 8.8, 5.5 Hz, 1H), 3.67 - 3.51 (m, 14H), 3.45 - 3.32 (m, 4H), 2.80 (t, *J* = 6.7 Hz, 2H), 2.69 (s, 3H), 2.45 (s, 3H), 1.83 (p, *J* = 6.5 Hz, 2H), 1.75 (p, *J* = 6.3 Hz, 2H), 1.70 (s, 3H).  19F NMR (376 MHz, Methanol-*d*4) an insignificant signal was observed at ä -77.42 ppm. HRMS (QTof) C29H39ClN6O4S, [M+H]+ = (calculated) 603.2520, (measured) 603.2504. 

**Synthesis of compound 18:** Biotin-NHS (46 mg, 0.135 mmol) was added to a solution of compound **17** (70 mg, 0.098 mmol) and DIEA (42.6 µl, 0.244 mmol) in DMF (Volume: 550 µl). The reaction was stirred at rt overnight under a nitrogenous atmosphere. The reaction was diluted in methanol (total volume = 3.0 mL) and was passed through a syringe-driven, 0.2 um filted. The filtrate was purified by preparative SFC (Method M1104) to afford WONGMAC-003-EXP080-001 (62 mg, 0.074 mmol, 76 % yield) 

1H NMR (400 MHz, DMSO-d6) δ 8.22 (t, J = 5.6 Hz, 1H), 7.77 (t, 1H, *J* = 5.5 Hz), 7.51 - 7.49 (m, 2H), 7.43 - 7.41 (m, 2H), 6.45 (br s, 1H), 6.38 (br s, 1H), 4.50 (dd, 1H, *J* = 7.9, 6.2 Hz), 4.30 (dd, 1H, *J* = 7.7, 5.0 Hz), 4.14 - 4.10 (m, 1H), 3.53 - 3.51 (m, 4H), 3.48 (d, J = 3.2 Hz, 4H), 3.49 - 3.45 (m, 4H), 3.44 (t, 2H, *J* = 6.7 Hz), 3.38 (t, J = 6.7 Hz, 2H), 3.36 (s, 3H), 3.25 (dd, 1H, *J* = 15.0, 8.1 Hz), 3.20 (d, J = 6.1 Hz, 1H), 3.18 (s, 1H), 3.16 (s, 1H), 3.15 - 3.03 (m, 4H), 2.89 (s, 1H), 2.81 (dd, 1H, *J* = 12.4, 5.1 Hz), 2.73 (s, 1H), 2.60 (s, 3H), 2.57 (d, 1H, *J* = 12.4 Hz), 2.41 (s, 3H), 2.04 (t, 2H, *J* = 7.4 Hz), 1.72 - 1.66 (m, 2H), 1.62-1.53 (m, 6H), 1.54 - 1.41 (m, 3H), 1.36 - 1.20 (m, 3H). 

13C NMR (101 MHz, DMSO) δ 171.82, 169.37, 162.98, 162.64, 162.26, 155.04, 149.78, 136.68, 135.17, 132.22, 130.65, 130.08, 129.76, 129.49, 128.44, 69.71, 69.50, 68.03, 67.97, 60.97, 59.11, 55.40, 53.83, 48.55, 37.57, 35.75, 35.71, 35.64, 35.15, 30.72, 29.41, 29.36, 28.18, 27.99, 25.26, 14.04, 12.65, 11.27. 

HRMS C39H53ClN8O6S2: [M+H]+ = (calculated) 829.3293, (measured) 829.3268. 
